# Selections

**Published:** 1895-06

**Authors:** 


### Selections.

### DIIODOFORM.

*La Presse Médicale* for February 24 contains an article by M. L. Maquenne, in which he remarks that, owing to the strong and persistent odor of iodoform, a large number of physicians have been obliged to do away with it in their private practice. For a long time they have searched for a drug to replace it which would produce the same effects, and without the disagreeable odor; but it has been difficult to find one with an organic combination comparable to iodoform with regard to the abundance of iodine it contains and to its physiological action. Diiodoform, which is not to be confounded with the deodorized iodoform of commerce, is entirely satisfactory in this respect. It is a definite iodide of carbon, which answers to the formula $C_2I_4$; it is derived from ethylene or olefiant gas, and should be called, according to the rules of chemical nomenclature, periodized ethylene. It will be seen that it differs from iodoform, $CHI_3$, in containing no hydrogen and containing twice as much carbon; theoretically, it may be considered as a product of the condensation of iodoform in which two particles are united with loss of hydriodic acid. This, as well as its known physiological properties, has caused it to be commonly known as diiodoform. Its centesimal composition is extremely like that of ordinary iodoform, as will be seen by the following comparison:

| | Iodoform. | Diiodoform. |
|---|---|---|
| Iodine | 96.70 | 95.19 |
| Carbon | 3.05 | 4.81 |
| Hydrogen | 0.25 | 0.00 |
| | 100.00 | 100.00 |

The quantity of iodine is very nearly the same, exceeding greatly that contained in iodol and aristol, which are sometimes used instead of iodoform. This explains the analogy of the action of these two substances and their undisputed superiority over all other antiseptics having iodine for their base. Diiodoform is a yellow substance, almost entirely odorless in an ordinary temperature, which melts at 377.6° F., and becomes decomposed into its elements, carbon and iodine, under 392° F.; it is perceptibly volatile when heated, and can be sublimed. Completely insoluble in water, slightly soluble in alcohol, diiodoform is easily dissolved in carbon disulphide, in chloroform, in benzene, and in the majority of hydrocarbons, and it is deposited in the form of beautiful prismatic needles, which are pulverized for use like iodoform crystals. Experience has shown that it is well borne by the stomach, and that it is practically non-poisonous. With regard to its germicidal action, it is the same as that of iodoform,—*i.e.*, rather weak.—*New York Medical Journal.*

